# Secondary Primary Carcinoma Arising on the Flap Skin in the Oral Cavity—Case Series

**DOI:** 10.3390/diseases12120324

**Published:** 2024-12-12

**Authors:** Boris Kos, Dinko Martinovic, Danko Muller, Iva Markota, Zoran Karlovic, Josko Bozic, Emil Dediol

**Affiliations:** 1 Department of Maxillofacial Surgery, University Hospital of Dubrava, 10000 Zagreb, Croatia; 2 Department of Maxillofacial Surgery, University Hospital of Split, 21000 Split, Croatia; 3 Department of Pathology, University Hospital of Dubrava, 10000 Zagreb, Croatia; 4 School of Dental Medicine, University of Zagreb, 10000 Zagreb, Croatia; 5 Department of Pathophysiology, School of Medicine, University of Split, 21000 Split, Croatia

**Keywords:** oral squamous cell carcinoma, secondary primary carcinoma, head and neck

## Abstract

**Background:** Oral squamous cell carcinoma (OSCC) causes considerable morbidity and mortality rates, posing a major global health burden. The management of the OSCC is multidisciplinary, but still the gold standard is surgical resection and reconstruction of the postablative defect. The appearance of secondary primary OSCC is not uncommon; however, it is quite rare that it appears on the skin of the flap that was used for reconstruction during the previous surgical therapy. **Methods:** We present three cases in which a secondary primary OSCC appeared on the skin of two radial forearm free flaps and two on regional pectoralis major flaps. **Results:** Our case series show that, although relatively rare, there is a chance of a secondary primary tumor on the flap used for intraoral reconstruction after the first oncological reconstruction. According to the latest and available literature, there is still no explanation of the underlying mechanism that leads to this occurrence. **Conclusions:** The learning point of this case series should be that, aside from the neck metastasis or recurrence of the primary oral cancer, the clinicians should also bear in mind that the flap itself should be physically examined in detail.

## 1. Introduction

Oral squamous cell carcinoma (OSCC) is the sixth most widespread malignancy in the world and the most frequent malignancy among head and neck cancers [1]. Due to its sensitive location as well as considerable morbidity and mortality rates with which it is associated, OSCC is a major global health burden. While there are many etiological causes that contribute to the development of OSCC, tobacco and alcohol remain the most important risk factors [2,3].

In recent decades there were certain advances regarding the management of OSCC, especially regarding the surgical reconstruction of the post-excisional defect, but still the primary gold standard for the treatment of OSCC is the radical resection along with the dissection of the cervical lymph nodes [4]. Depending on the staging of the disease, usually an adjuvant chemoradiotherapy follows after the surgical management. According to the literature, the 5-year survival rate of OSCC is moving from 40% to 70%, depending on the author [5,6,7,8].

The goal of post-excisional defect reconstruction using regional or, more recently, free flaps is to recover the integrity and functionality of the oral cavity. Even though these flaps have skin, which is not in its normal surrounding in the oral cavity, some studies are suggesting that this skin undergoes “mucosalization”, a process in which it gets thicker, loses its stratum corneum, and reduces its pilosebaceous units [9,10,11]. However, this topic is still controversial, and further studies are needed to address this issue.

The appearance of a secondary primary OSCC on the skin from previous regional/free flaps is a very rare but possible occurrence [12,13]. There are several proposed explanations for this occurrence: (1) metaplasia of the skin due to chronic inflammation in the oral cavity; (2) colonization of the skin by the cells of adjacent mucosa that have previously been genetically altered and predisposed to the development of cancer; (3) continuation of previous bad habits, such as tobacco and alcohol consumption, which leads to a new primary cancer [14].

In this report, we present three cases of OSCC which occurred on the skin of two radial forearm free flaps and two regional pectoralis major flaps.

## 2. Case I

In 1999, a 40-year-old male patient was referred to our department due to intraoral pain. He had a history of tobacco and alcohol consumption. Upon physical examination, an ulceration was observed on the right buccal mucosa. Radiological finding showed a pathological necrotic lymph node at level II of the right side of the neck. The histopathological report of the biopsy confirmed an SCC, and the staging of the cancer was T2N1M0. The primary management was the resection of the cancer with an en block selective neck dissection of levels I, II, and III. The defect was reconstructed with a fasciocutaneous radial forearm free flap. The postoperative course was uneventful, except for a minor neck skin infection, and the patient later underwent adjuvant radiotherapy. The histopathological report showed clear surgical margins and confirmed a metastasis in the lymph node at level II.

Seven years later, on a regular follow-up, a new primary tumor was discovered in the center of the radial forearm flap skin. It was decided to surgically treat the tumor; hence, it was widely excised, and the defect was reconstructed with another radial forearm free flap. Histopathology specimens showed evidence that a new SCC arose directly from the center of the flap skin, while it did not spread to underlying tissue or adjacent mucosa. The histopathological report showed that surgical margins were negative for cancer. 

In September 2008, during another regular follow-up, an enlarged lymph node on the right side of the neck was noticed. Radiological findings showed a pathological necrotic lymph node in region IV of the right side of the neck as well as suspicious lymph nodes in the paratracheal and superior mediastinal regions. After FNA, cytological findings confirmed metastasis in the superior mediastinal region. A complete radical neck dissection of the right side of the neck was carried out in conjunction with a dissection of the paratracheal and superior mediastinal lymph nodes. Postoperative course was uneventful, and afterwards the patients underwent radiotherapy of the lower portion of the neck and superior mediastinum. 

In 2009, the patient noticed a painful lump in the right axilla. After FNA, a cytological report confirmed a metastatic SCC. Complete dissection of his right axilla was performed with subsequent radiotherapy afterwards. At the moment, the patient is well and without any evidence of the disease.

## 3. Case II

In 1998, a 32-year-old male patient was referred to our department due to major intraoral pain. He had a history of smoking and alcohol consumption. On physical examination, a large ulceration was observed on the left buccal mucosa with an extension on the corpus of the mandible. Radiological finding did not show any evident neck metastasis. Histopathological report of the biopsy confirmed SCC, and the staging of the tumor was T4aN1M0. The primary management was an en block resection of the tumor along with the resection of the mandible and radical neck dissection. The defect was reconstructed using a regional myocutaneous pectoralis major flap. The postoperative course was uneventful, and postoperative radiotherapy was administered eight weeks later. 

In September 2007, on a regular check-up, an ulceroinfiltrative lesion on the skin of the pectoralis major flap was observed. A biopsy was taken, and the histopathological report confirmed squamous cell carcinoma on the skin of the flap. The patient underwent reexcision of the cancer, and the histopathology report of the specimen showed no evidence of residual carcinoma on the margins. At the moment the patient is well, with no evidence of the disease.

## 4. Case III

We present a case of a 76-year-old patient with a 20-year smoking history and no history of alcohol abuse. The patient initially underwent partial maxillectomy for early-stage oral squamous cell carcinoma (OSCC) of the right maxillary gingiva in 1994. Pathohistological analysis revealed pT2 OSCC, and no adjuvant therapy was required. Regular follow-up was maintained for the first 6 years. 

In 2012, the patient presented with an ulcerated lesion in the region of the previous maxillectomy, infiltrating adjacent cheek soft tissue. The patient experienced difficulty with mouth opening and displayed bloody discharge. A biopsy revealed a new primary OSCC. Multislice computed tomography (MSCT) confirmed spread in the masticatory space and two enlarged lymph nodes at levels I and II on the right side of the neck, staging the disease as cT4bN2b. A radical maxillectomy was performed, including resection of the masticatory space, segmental resection of the mandibular ramus and infiltrated skin, and a modified radical neck dissection with preservation of the internal jugular vein. Reconstruction of the palate and buccal mucosa was achieved with a regional pectoral muscle flap, while bone was reconstructed using a titanium mesh and the skin with a local cervicofacial flap. Pathohistological analysis revealed pT4aN0 OSCC. Adjuvant radiotherapy was administered (66 Gy/33 fractions). One year later, reconstruction of the maxillary and adjacent soft tissue defect was undertaken due to titanium mesh exposure. Midfacial bone and facial skin were reconstructed with a latissimus dorsi-scapula free flap. Throughout follow-up, the patient was evaluated every 2–3 months for the first 2 years, every 6 months for the next 4 years, and then annually. Each follow-up included a regular physical examination, with an ultrasound of the neck conducted 1–2 times per year and imaging (CT or MRI) of the head, neck, and chest repeated annually. 

In 2021, the patient presented with new primary synchronous OSCC on the gingiva of the right mandible spreading to adjacent skin (cT4a) and one in the left sublingual region (cT3). The neck was clinically negative. Resection of oral synchronous cancers with segmental mandibular resection and resection of involved skin en bloc with elective left-side selective neck dissection (I-IV) was performed. Bone and oral mucosa defects were reconstructed with an osteocutaneous radial forearm free flap from the patient’s left arm, and the involved skin was reconstructed with an axial deltopectoral flap. Pathohistological analysis revealed two synchronous OSCCs: one on the right gingiva (verrucous OSCC, staged pT4a) and one sublingual OSCC (staged pT3). The neck was negative for metastases (N0). Adjuvant radiotherapy was administered (66 Gy/33 fractions). 

One year later, 29 years after the initial operation, the patient clinically presented with two synchronous verrucous OSCCs arising in the center of the right buccal mucosa (previously reconstructed with a regional flap of the major pectoral muscle) and the center of the sublingual mucosa (previously reconstructed with a radial forearm free flap). The patient underwent the resection of the two oral carcinomas with reconstruction using a rectus abdominis free flap. 

**Figure 1:**
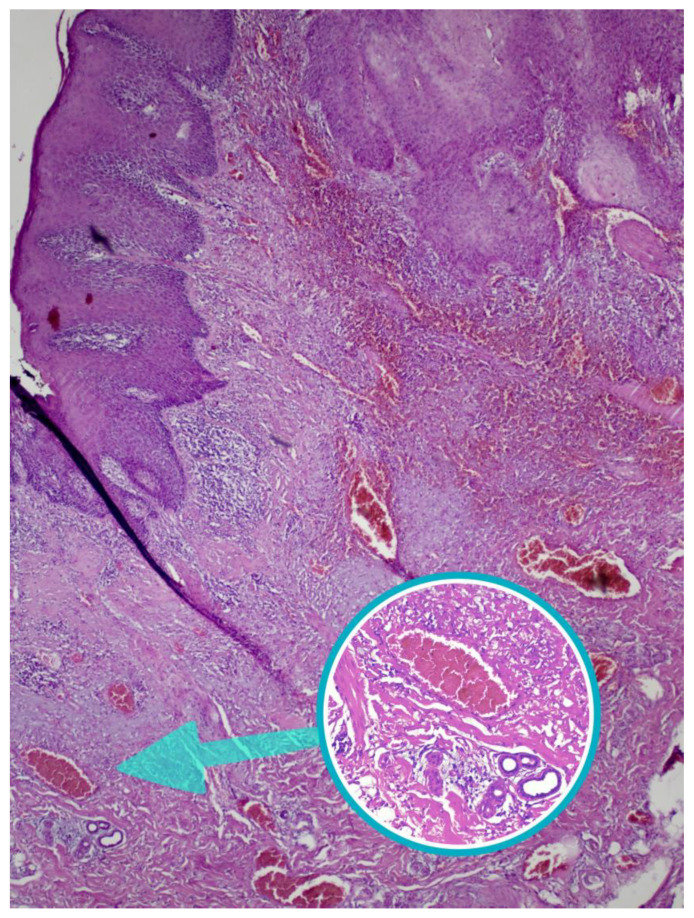
Microscopic image of the tumor where overlying skin and adnexal structures are visible. The enlarged circle is showing the apocrine and eccrine glands (HE 200×).

The histopathological report showed that the material is a part of the tongue, the base of the oral cavity, and the buccal mucosa measuring 9.5 × 5 cm; there are two white patches, partly ulcerated, measuring 3 × 1.5 × 1 cm and 4.2 × 2.5 × 0.5 cm. Histologically, the tumor is comprised of islands and cords of well-differentiated squamous epithelial cells. It consists of areas of extensive keratinization forming multiple scales and keratin pearls; tumor cells show minimal pleomorphism. Among the described islands and cords, there is an abundant desmoplastic connective stroma permeated with a mononuclear inflammatory infiltrate. No perineural invasion was noted. The tumor does not reach the resection margins; the closest resection margin is in the buccal mucosa and measures 4 mm. Remains of adnexal structures (referring to the flap skin) are visible towards the buccal mucosa (Figure 1). The findings suggest that the tumor is a well-differentiated squamous cell carcinoma.

## 5. Discussion

While the occurrence of secondary primary OSCC is not uncommon, especially in the oral region, because of the previous prolonged effect of tobacco and alcohol consumption in the majority of these patients, a secondary primary carcinoma on the flap used in a previous surgical management is particularly rare. A recent systematic review reported that there were only 27 cases described in the literature [12]. All of the cases were in the oral cavity, and all of the secondary primary OSCC were located in the center of the flap skin with clear margins. Furthermore, there was a recent cohort study that included 21 patients with potentially malignant disorders or skin cancer on the intraoral flap skin [15]. They conducted an immunohistochemical study with a focus on p53 and p16. Their results showed that these second primary tumors had a relatively low altered p53 and a relatively high p16 positivity.

The pathophysiology underlying cancer development on skin flaps in the oral cavity is a subject of controversy and ongoing discussion [12,15]. One theory posits that neoplastic cells migrate from altered mucosa to the skin flap, a phenomenon known as the “Borst–Jadasson phenomenon”, subsequently progressing into cancer [16,17]. This process is further elucidated by the field cancerization theory [13,18,19]. Moreover, Foschini et al. discovered that neoplastic lesions originating on skin flaps in the oral cavity share the same clone and genetics as the primary oral squamous cell carcinoma (OSCC) [20]. Hence, a possible explanation is that the cancer cells migrate from the surrounding mucosa to the flap.

Conversely, in a retrospective study, Eguchi et al. argue that cancers on the skin of flaps exhibit low TP53 mutations and higher p16 positivity compared to OSCC, suggesting they should be considered a distinct entity [21]. They propose that exposure of the skin to a new environment induces carcinogenesis through chronic irritation and inflammatory reactions, resulting in the formation of skin cancer within the oral cavity [22]. Furthermore, Petruzzi et al. assert that skin exposure to new stressors, such as saliva, altered pH, and oral flora, may contribute to carcinogenesis. They draw parallels with the development of adenocarcinoma in Barrett’s esophagus [12]. Additionally, Eguchi et al. compare the mechanism of carcinogenesis on skin flaps due to chronic irritation with the carcinogenesis commonly observed in burn scar tissue [15,23,24]. Therefore, another possibility is that the skin, due to new and “alien” environmental factors in the oral cavity, undergoes inflammatory reactions that could trigger an emergence of a new primary cancer. Nevertheless, determining the correctness of either theory, or if both theories exert some influence, requires further research.

Another possible explanation of the secondary primary cancer emergence on the flap skin is the process called mucozalization [11]. Macroscopically, the skin of the flaps after a prolonged time in the oral cavity starts to appear similar to the surrounding mucosa [25]. Moreover, several histological articles have suggested that there is an alteration of the skin histology after several years in the oral cavity [11,26]. These histological changes are characterized by inflammatory infiltration, reduction of the stratum corneum thickness, and loss of skin appendages. However, this theory is severely challenged, and some authors have proposed that actually the chronic candida infection due to the “alien” environment is the reason behind the histological changes of the flap skin [27]. Nevertheless, it seems that the skin gradually undergoes some changes. The histopathological reports of our presented cases, especially Case III, which underwent flap reconstruction almost 30 years ago, showed that there were very mild or no changes in the structure of the flap’s skin and that the adnexal structures were well preserved after a long period of time. 

In conclusion, our case series shows that, although relatively rare, there is a chance of a secondary primary tumor on the flap used for intraoral reconstruction after the first oncological reconstruction. According to the latest and available literature, there is still no explanation of the underlying mechanism that leads to this occurrence. The learning point of this case series should be that, aside from the neck metastasis or recurrence of the primary oral cancer, the clinicians should also bear in mind that the flap itself should be physically examined in detail. Moreover, the underlying pathological mechanism of this occurrence should be further researched for a better understanding and, consequently, better management of these patients.

## Data Availability

Data are contained within the article.

## References

[1] Sasahira T., Kirita T. (2018). Hallmarks of Cancer-Related Newly Prognostic Factors of Oral Squamous Cell Carcinoma. Int. J. Mol. Sci..

[2] Capote-Moreno A., Brabyn P., Muñoz-Guerra M.F., Sastre-Pérez J., Escorial-Hernandez V., Rodríguez-Campo F.J., García T., Naval-Gías L. (2020). Oral squamous cell carcinoma: Epidemiological study and risk factor assessment based on a 39-year series. Int. J. Oral Maxillofac. Surg..

[3] Nokovitch L., Maquet C., Crampon F., Taihi I., Roussel L.M., Obongo R., Virard F., Fervers B., Deneuve S. (2023). Oral Cavity Squamous Cell Carcinoma Risk Factors: State of the Art. J. Clin. Med..

[4] Caruntu A., Caruntu C. (2022). Recent Advances in Oral Squamous Cell Carcinoma. J. Clin. Med..

[5] Ferreira A.K., Carvalho S.H., Granville-Garcia A.F., Sarmento D.J., Agripino G.G., Abreu M.H., Melo M.C., Caldas A.D., Godoy G.P. (2021). Survival and prognostic factors in patients with oral squamous cell carcinoma. Med. Oral Patol. Oral Cir. Bucal.

[6] Taghavi N., Yazdi I. (2015). Prognostic factors of survival rate in oral squamous cell carcinoma: Clinical, histologic, genetic and molecular concepts. Arch. Iran. Med..

[7] Ahmad P., Nawaz R., Qurban M., Shaikh G.M., Mohamed R.N., Nagarajappa A.K., Asif J.A., Alam M.K. (2021). Risk factors associated with the mortality rate of oral squamous cell carcinoma patients: A 10-year retrospective study. Medicine.

[8] Bakshi J., Kaur N., Tiwana H., Verma R.K., Panda N.K., Patro S.K. (2023). Survival Analysis of Oral Squamous Cell Carcinoma Patients Attending Tertiary Care Centre of North India. Indian J. Surg. Oncol..

[9] Woolgar J.A., Triantafyllou A. (2009). Histological changes in intra-oral skin flaps. Head Neck Oncol..

[10] Choi J.W., Kim K.N., Park E.J., Eom J.S., Hong J.P., Park H.N., Park C.S., Kim S.Y., Nam S.Y., Choi S.H. (2014). Analysis of morphological and histologic changes in intraoral fasciocutaneous free flaps used for oropharyngeal reconstruction. Ann. Plast. Surg..

[11] Shibahara T., Noma H., Takeda E., Hashimoto S. (2000). Morphologic changes in forearm flaps of the oral cavity. J. Oral Maxillofac. Surg..

[12] Petruzzi G., De Bonis T., Manciocco V., Pichi B., Rosati V., Iocca O., Sedran L., Moretto S., Campo F., De Virgilio A. (2023). Second primary carcinoma arising on a flap: A new primary or a relapse?. Acta Biomed..

[13] Ho A.C., Fan P.Y., Shek T.W., Ho D., Wei W.I. (2011). Second primary squamous cell carcinoma arising in the skin of a pectoralis major myocutaneous flap 12 years after floor of mouth reconstruction. Eur. Arch. Otorhinolaryngol..

[14] Monnier Y., Pasche P., Monnier P., Andrejevic-Blant S. (2008). Second primary squamous cell carcinoma arising in cutaneous flap reconstructions of two head and neck cancer patients. Eur. Arch. Otorhinolaryngol..

[15] Eguchi K., Kobayashi K., Honma Y., Ryo E., Sakyo A., Yokoyama K., Watanabe T., Aihara Y., Sakai A., Matsumoto Y. (2023). Clinical and pathological features of second primary neoplasms arising in head and neck reconstructive skin flaps. Sci. Rep..

[16] Helm K.F., Helm T.N., Helm F. (1994). Borst-Jadassohn phenomenon associated with an undifferentiated spindle cell neoplasm. Int. J. Dermatol..

[17] Braakhuis B.J., Leemans C.R., Brakenhoff R.H. (2004). A genetic progression model of oral cancer: Current evidence and clinical implications. J. Oral Pathol. Med..

[18] Chang J.H., Wu C.C., Yuan K.S., Wu A.T.H., Wu S.Y. (2017). Locoregionally recurrent head and neck squamous cell carcinoma: Incidence, survival, prognostic factors, and treatment outcomes. Oncotarget.

[19] Sung H., Ferlay J., Siegel R.L., Laversanne M., Soerjomataram I., Jemal A., Bray F. (2021). Global Cancer Statistics 2020: GLOBOCAN Estimates of Incidence and Mortality Worldwide for 36 Cancers in 185 Countries. CA Cancer J. Clin..

[20] Foschini M.P., Morandi L., Marchetti C., Cocchi R., Eusebi L.H., Farnedi A., Badiali G., Gissi D.B., Pennesi M.G., Montebugnoli L. (2011). Cancerization of cutaneous flap reconstruction for oral squamous cell carcinoma: Report of three cases studied with the mtDNA D-loop sequence analysis. Histopathology.

[21] Kobayashi K., Yoshimoto S., Matsumoto F., Ando M., Murakami N., Omura G., Fukasawa M., Matsumoto Y., Matsumura S., Akamatsu M. (2019). All-Exon TP53 Sequencing and Protein Phenotype Analysis Accurately Predict Clinical Outcome after Surgical Treatment of Head and Neck Squamous Cell Carcinoma. Ann. Surg. Oncol..

[22] Johnson F.E., Chang P.K., Huvos A.G., Strong E.W. (1983). Neoplastic changes in transposed deltopectoral skin. Arch. Otolaryngol..

[23] Mousa A.K., Elshenawy A.A., Maklad S.M., Bebars S.M.M., Burezq H.A., Sayed S.E. (2022). Post-burn scar malignancy: 5-year management review and experience. Int. Wound J..

[24] Kowal-Vern A., Criswell B.K. (2005). Burn scar neoplasms: A literature review and statistical analysis. Burns.

[25] Badran D., Soutar D.S., Robertson A.G., Reid O., Milne E.W., McDonald S.W., Scothorne R.J. (1998). Behavior of radial forearm skin flaps transplanted into the oral cavity. Clin. Anat..

[26] Eliachar I., Sebek B.A., Levine S., Tucker H.M. (1985). Histologic changes in skin implanted into the larynx and trachea by myocutaneous flap reconstruction. Otolaryngol. Head Neck Surg..

[27] Khan A.L., Cloke D.J., Hodgkinson P.D., McLean N.R., Soames J.V. (2001). Do intraoral radial forearm free flaps re-mucosalise and is candida infection relevant?. Br. J. Plast. Surg..

